# Acute acalculous cholecystitis due to breast cancer metastasis to the cystic duct

**DOI:** 10.1186/s40792-016-0239-1

**Published:** 2016-10-12

**Authors:** Masakazu Hashimoto, Kei Koide, Michinori Arita, Koji Kawaguchi, Masakazu Tokunaga, Yoshihiro Mikuriya, Toshiyuki Iwamoto

**Affiliations:** 1 Department of Surgery, Chuden Hospital, 3-4-27 Otemachi, Naka-Ku, Hiroshima 730-8562 Japan; 2 Department of Pathology, Chuden Hospital, 3-4-27 Otemachi, Naka-Ku, Hiroshima 730-8562 Japan

**Keywords:** Acute cholecystitis, Biliary metastasis, Breast cancer, Late recurrence

## Abstract

**Background:**

Acute acalculous cholecystitis (AAC) is a relatively rare disorder of the gallbladder. Breast cancer recurrence more than 10 years after curative surgery is also infrequent.

**Case presentation:**

Here, we report a case of a 59-year-old woman who presented with right flank pain. Her medical history included a lumpectomy for cancer of the left breast 12 years prior. Laboratory tests showed a severe inflammatory reaction and mild liver function abnormalities. Ultrasonography and computed tomography revealed an enlarged gallbladder and inflammation of the surrounding tissues; however, no gallstone was present. She was diagnosed with AAC. We performed an emergency laparoscopic cholecystectomy, and histopathological examination revealed a poorly differentiated adenocarcinoma in the cystic duct. Both metastatic and primary tumor cells were positive for estrogen and progesterone receptors on immunohistochemistry. The final pathological diagnosis was acute cholecystitis due to breast cancer metastasis to the cystic duct.

**Conclusion:**

Although AAC secondary to metastatic breast cancer is rare, it should be included in the differential diagnosis for abdominal pain in patients with a previous history of breast cancer.

## Background

Acute acalculous cholecystitis (AAC) is characterized by gallbladder inflammation without cystic duct obstruction due to gallstones. It is clinically indistinguishable from acute calculous cholecystitis (ACC). AAC accounts for 2–12 % of acute cholecystitis cases [1–3]. Most cases of AAC are related to surgery, total parental nutrition, and prolonged fasting [4, 5]; AAC caused by metastases to the gallbladder is relatively infrequent [6].

Breast cancer has a high recurrence rate, and recurrences tend to occur within 5 years of surgery. Recurrences after more than 10 years of disease-free survival are rare, although they are still commoner than in other cancers such as colon and gastric cancer [7–9].

We report a case of AAC secondary to metastatic breast cancer. This was discovered incidentally after cholecystectomy in a patient who had 12 years of disease-free survival.

## Case presentation

**Fig. 1:**
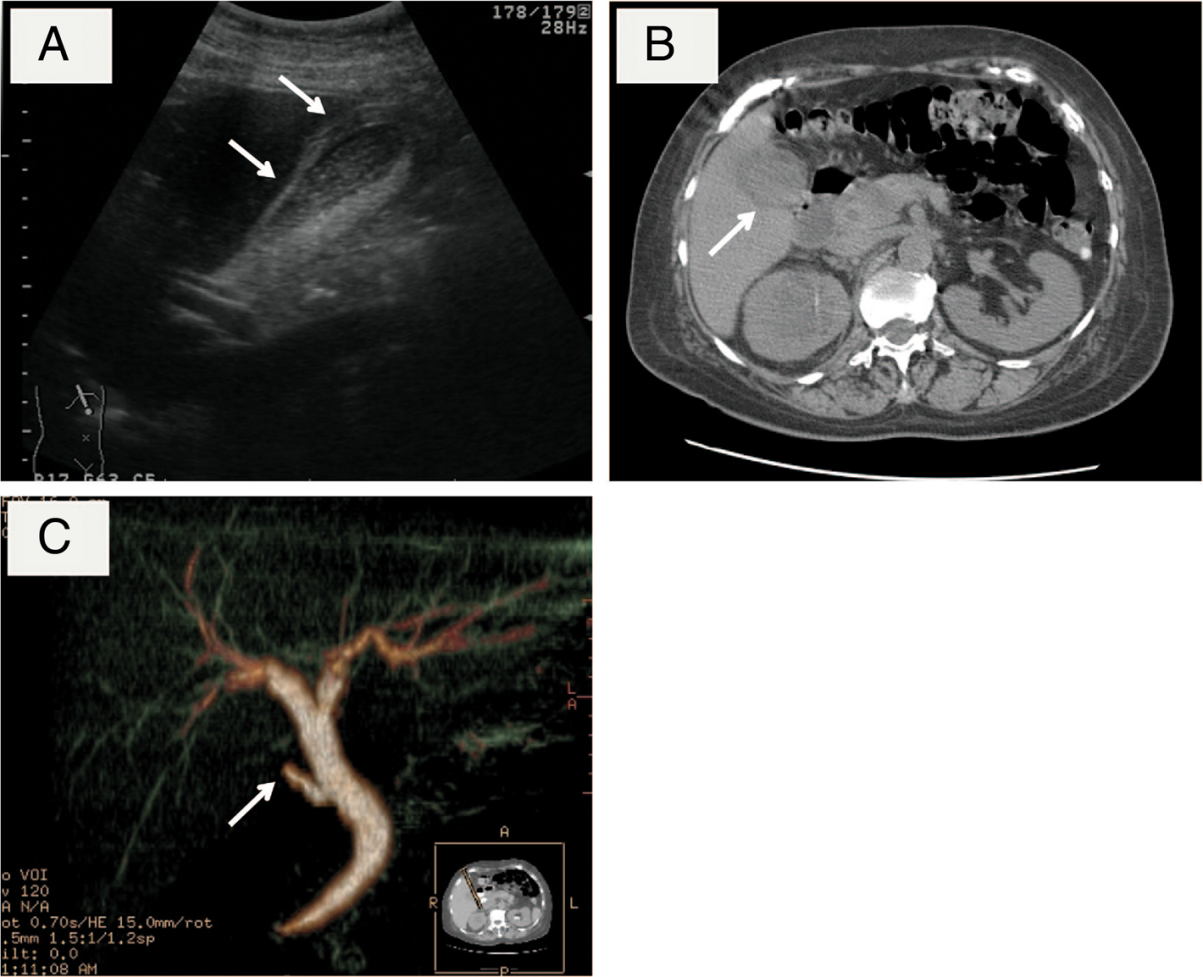
**a** US showed thickening of the gallbladder wall and subserosal edema. **b** CT showed the enlarged gallbladder and the thickened gallbladder wall. **c** DIC-CT showed no gallstones in the common bile duct and an interruption of cystic duct

A 59-year-old woman presented to our hospital complaining of right flank and epigastric pain. An examination of the abdomen revealed tenderness in the right upper quadrant and positive Murphy’s sign. Her laboratory test results were as follows: white blood cell count, 13,600/mm^3^; hemoglobin, 8.2 g/dL; platelet count, 20.6 × 10^4^/mm^3^; aspartate aminotransferase, 45 IU/L; alanine aminotransferase, 61 IU/L; total bilirubin, 1.0 mg/dL; and C-reactive protein, 26.2 mg/dL. The levels of carcinoembryonic antigen (CEA) and carbohydrate antigen 19-9 were 6.3 ng/mL and 224.9 U/mL, respectively. Abdominal ultrasonography (US) revealed a thickened gallbladder wall and subserosal edema. Computed tomography (CT) also revealed an enlarged gallbladder and pericholescystic fluid collection (Fig. 1b). However, stones were not observed in the gallbladder or cystic duct, and the cause of the acute cholecystitis could not be identified. Drip-infusion cholangiography-CT (DIC-CT) confirmed the lack of patency of the cystic duct and showed no gallstones in the common bile duct (Fig. 1c).

The patient’s medical history included a lumpectomy of invasive ductal carcinoma of the left breast and negative sentinel lymph node (pT1c/pN0) 12 years before. She had been followed up with US, CT, and fluorine-18-fluorodeoxyglucose positron-emission tomography (FDG-PET) imaging for 10 years, and no recurrence had been observed as of her last follow-up.

**Fig. 2:**
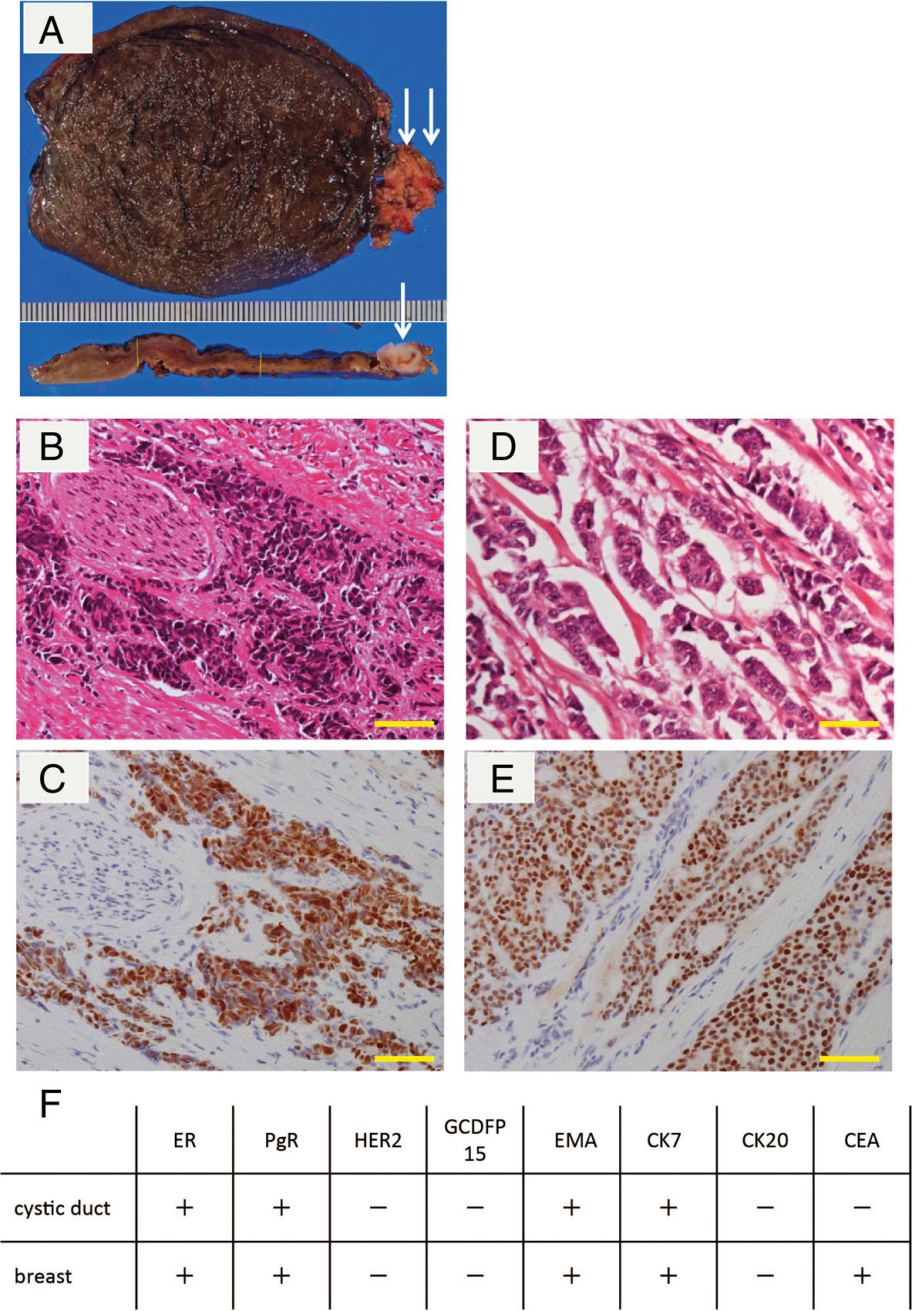
**a** Macroscopically, the gallbladder mucosa was necrotic and black. The cystic duct and gallbladder bladder were thickened and white. **b** Microscopic examination revealed a poorly differentiated adenocarcinoma in the cystic duct. *Scale bar* is 50 μm. **c** Immunohistochemical examination revealed that the tumor cells were positive for ER. *Scale bar* is 50 μm. **d** The primary breast cancer was an invasive ductal carcinoma. *Scale bar* is 50 μm. **e** Immunohistochemical examination revealed that the tumor cells of the primary breast cancer were positive for ER. *Scale bar* is 50 μm. **f** Comparison between metastatic tumor cells (cystic duct) and primary breast cancer cells on immunohistochemical examination

The patient was diagnosed with AAC and underwent a laparoscopic cholecystectomy without any complications. Macroscopically, the gallbladder mucosa appeared necrotic. Histopathological examination revealed a poorly differentiated adenocarcinoma in the cystic duct and gallbladder neck. On immunohistochemical examination, the tumor cells were positive for estrogen and progesterone receptors (ER and PR). The tumor cells were also positive for cytokeratin-7 and epithelial membrane antigen and were negative for human epidermal growth factor receptor 2 (HER2), gross cystic disease fluid protein-15, and cytokeratin-20. These results were similar to the immunohistochemical findings from the primary breast cancer (Fig. 2f). Accordingly, the pathological diagnosis of metastatic breast cancer was made. One month after cholecystectomy, an FDG-PET scan revealed abdominal para-aortic lymph node metastases and a lumbar vertebra metastasis. The patient was treated with chemotherapy and hormone therapy, and she died 5 years later (17 years later after breast surgery).

### Discussion

AAC is associated with a higher mortality rate and has a worse prognosis than ACC [4, 10, 11]. Most cases of AAC occurs in critically ill patients and are related to surgery, burns, severe trauma, bacterial sepsis, shock, congestive heart failure, total parenteral nutrition, and prolonged fasting [4, 5]. AAC is also associated with gallbladder cancer and bile duct cancer [12, 13]. Ida et al. reported a 6.9 % incidence of AAC in gallbladder cancer [14]. In contrast, cystic duct cancer is extremely rare and is not usually diagnosed prior to cholecystectomy [15]. Thickening of the gallbladder neck and cystic duct walls in the context of AAC can suggest the presence of cancer. Endoscopic ultrasonography and cytodiagnosis from endoscopic naso-gallbladder drainage may be useful in identifying this thickening [16–18].

Metastasis to the gallbladder is very rare. Metastases originating from malignant melanomas, and lung, renal, pancreatic, and colorectal cancers have been reported [6, 19]. Breast cancer metastasis to the gallbladder is rare and our literature search revealed only 25 cases. Only one case of cystic duct metastasis had been reported [20]. In this case, cystic duct metastasis occurred after metastases to both lobes of the liver, and right-supraclavicular node, which was found 3 years after mastectomy. Acalculous cholecystitis was indicated based on the clinical finding of obstruction of the cystic duct by liver metastasis. Laparotomy revealed a solitary metastatic deposit surrounding the proximal cystic duct. In our case, prior to the cholecystectomy, the suspicion of breast cancer metastasis to the cystic duct was low because the patient had remained cancer-free for over 10 years.

Breast cancer is the commonest form of malignancy in females. Postoperative recurrence occurs in approximately 30 % of cases [21]. The commonest sites of recurrence are the bone, lung, and liver [7–9]. Owing to advances in chemotherapy and endocrine therapy, the prognosis for breast cancer has improved over the years. Despite this, many patients continue to experience disease recurrence. Recurrences tend to occur within the first 5 years after surgery; late recurrences after more than 10 years are very uncommon [7, 9, 22]. Late recurrences have been found to affect the bone and lung in 33.3 % of patients, and the recurrence patterns of late and early recurrences were not found to be significantly different [22]. Lymph node metastases [23], ER-positive status [9], and HER2-negative status [24] are reported to be risk factors for late recurrence in breast cancer patients. In our case, the patient’s tumor cells were positive for ER and PR and negative for HER2 on immunohistochemical examination. Moreover, it was recently reported that extension of hormonal treatment to 10 years was useful for preventing recurrences in such patients [25]. On the other hand, post-relapse survival was significantly longer in patients with late recurrences than in patients with early recurrences [26, 27]. In the present case, after multiple metastases were diagnosed, the patient was treated with aromatase inhibitor therapy, bisphosphonate therapy, and chemotherapy such as paclitaxel and epirubicin, and she survived for 5 years.

In this patient, even if her breast cancer metastases were diagnosed before cholecystectomy, this knowledge might not have been useful in guiding the clinical decision-making process because multiple metastases were detected on FDG-PET only 2 weeks after the surgery. However, most cases of late breast cancer recurrence involve solitary tumors, which can be radically treated to improve patient survival [28]. As such, it is important to be able to identify AAC due to metastases from breast cancer recurrence prior to surgery.

## Conclusions

In conclusion, we have reported a case of AAC secondary to cystic duct metastasis from recurrent breast cancer. It is necessary to consider metastatic breast cancer as a cause of AAC in patients with a history of breast cancer.

## Consent

Written informed consent was obtained from the patient for publication of this case report and any accompanying images.

## Abbreviations

AAC: Acute acalculous cholecystitis
ACC: Acute calculous cholecystitis
CEA: Carcinoembryonic antigen
CT: Computed tomography
DIC-CT: Drip-infusion cholangiography computed tomography
ER: Estrogen receptor
FDG-PET: Fluorine-18-fluorodeoxyglucose positron-emission tomography
HER2: Human epidermal growth factor receptor 2
PR: Progesterone receptor
US: Ultrasonography
